# A De Novo Missense Variant in *TUBG2* in a Child with Global Developmental Delay, Microcephaly, Refractory Epilepsy and Perisylvian Polymicrogyria

**DOI:** 10.3390/genes14010108

**Published:** 2022-12-29

**Authors:** Salini Thulasirajah, Xueqi Wang, Erick Sell, Jorge Dávila, David A. Dyment, Kristin D. Kernohan

**Affiliations:** 1 Division of Neurology, Children’s Hospital of Eastern Ontario, Ottawa, ON K1H 8L1, Canada; 2 Children’s Hospital of Eastern Ontario Research Institute, Ottawa, ON K1H 8L1, Canada; 3 Department of Radiology, Children’s Hospital of Eastern Ontario, Ottawa, ON K1H 8L1, Canada; 4 Newborn Screening Ontario (NSO), Ottawa, ON K1H 8L1, Canada; 5 Department of Genetics, Children’s Hospital of Eastern Ontario, Ottawa, ON K1H 8L1, Canada

**Keywords:** polymicrogyria, tubulinopathy, exome sequencing

## Abstract

Polymicrogyria is a brain malformation characterized by excessive folding of the cortex. To date, numerous causes of polymicrogyria have been identified, including variants in the genes associated with tubulinopathies. Herein, we present a child with severe intellectual disability, refractory to treatment seizures, microcephaly and MRI findings consistent with polymicrogyria, closed-lip schizencephaly, periventricular heterotopia and a dysplastic corpus callosum. Exome sequencing identified a de novo missense variant in *TUBG2*, a gene not associated with human disease. The variant, NM_016437.3 c.747G>A p.(Met249Ile), is absent from available control databases and is predicated to be deleterious by in silico prediction programs. Laboratory studies show that cultured lymphoblasts derived from the patient grew significantly faster than controls. Recombinant protein was expressed (recombinant wild type and mutant TUBG2-FLAG) in 293T cells and lower levels of TUBG2 mutant compared with controls were observed. Furthermore, co-immuno-precipitation in cells transfected demonstrated that the TUBG2–GCP2 interaction is increased due to the MUT recombinant protein versus WT recombinant protein. In closing, this work provides preliminary evidence that *TUBG2* may represent a novel disease gene responsible for polymicrogyria.

## 1. Introduction

Polymicrogyria (PMG) is a brain malformation caused by the presence of excessive folding and abnormal laminations of the cortex [1]. PMG is one of the most common brain anomalies, accounting for 20% of cases of malformations of cortical development [2]. There is a wide clinical spectrum of comorbidities associated with PMG, with the most common being epilepsy (78%), global developmental delay (70%) and spasticity (51%) [2,3]. The generalized forms of PMG result in more severe symptoms than the bilateral, focal or unilateral forms, suggesting that the extent of PMG has a significant influence on the subsequent clinical manifestations [2,3]. PMG is known to have both genetic and non-genetic causes. Examples of non-genetic causes include hypoxia, hypoperfusion and congenital infections such as cytomegalovirus [4]. Genetic causes include both chromosomal abnormalities [5,6] and an expanding list of over 40 single-gene conditions [6,7]. Pathogenic variation in key cellular pathways, such as the mTORopathies, tubulinopathies and cobblestone dysplasias (α dystroglycanopathies and other including laminopathies and congenital disorders of glycosylation) represent a growing list of gene mutations that result in PMG [6]. However, it is important to note that while many genes have been described, a significant portion remains unidentified [6].

The α-tubulin and β-tubulin genes encode proteins, which form the structural components of microtubules, a part of the cytoskeleton that is critically important for neuronal migration and, when defective, the formation of PMG. Perhaps not surprisingly, pathogenic variants in many genes in this pathway are implicated in a number of neurological diseases, termed tubulinopathies [8,9]. The phenotypic spectrum of tubulinopathies include lissencephaly to asymmetric perisylvian PMG with dysmorphic basal ganglia, cerebellar vermis dysplasia and pontine hypoplasia [9].

Herein, we present an eight-year-old male with bilateral perisylvian polymicrogyria, who was found to have a de novo variant in *TUBG2* by exome sequencing. This gene has not yet been reported to be associated with human disease. Functional studies confirmed that this variant can impact protein function and thereby provides preliminary evidence for TUBG2 as a gene associated with PMG.

## 2. Material and Methods

### 2.1. Exome Sequencing and Analysis

For exome sequencing, exonic DNA was selected using the Agilent SureSelect CRE kit following the manufacturer’s instructions and sequenced on a NextSeq500. Read alignment, variant calling and annotation were performed as previously described for Care4Rare Canada projects, with a pipeline based on Burrows–Wheeler Aligner, Picard, ANNOVAR and custom annotation scripts [10,11,12,13]. The average coverage for the exomes was 121× for the affected individual and 104× and 119× for the mother and father, respectively, and 95% of the CCDS exons in all exomes were covered at >10×. Variants were disregarded if they were present at >1% in gnomAD or seen in more than 5 samples from our in-house database (∼2000 exomes previously sequenced from our cohorts). PCR and Sanger sequencing was used to validate the variants identified.

### 2.2. Expression of Recombinant TUBG2

TUBG2 wild type and variant (NM_016437.3 c.747G>A: p.(Met249Ile)) with a FLAG tag at the C terminus (TUBG2-FLAG) were cloned into pcDNA3.1 vector through the Gencript Mutation and Library Service. Approximately 1.5 to 3.5 × 105 293T cells were plated in 4 mL of DMEM medium with penicillin–streptomycin and G418 in each well of 6 well plates. After overnight culture, they were transfected with 5 μg of pcDNA3.1 plasmid expressing wild type or mutant TUBG2 using 3.75 μL of Lipofectamine 3000 (Invitrogen, Waltham, MA, USA). The cells were collected between 48 to 66 h post-transfection.

### 2.3. Western Blot

The patient cultures and control lymphoblasts were lysed in RIPA (50 mM Tris-HCl [pH 8.0], 150 mM NaCl, EDTA 5 mM, 1% NP-40, Sodium deoxycholate 0.5%, SDS 0.1% with protease inhibitors P8340 (Sigma, St. Louis, MO, USA) for 20 min on ice with random vortex. Approximately 20 μg of clarified lysate was resolved by the 10% TGX Stain-Free acrylamide gel (Bio-Rad, Hercules, CA, USA). The gel was imaged using the stain-free application on the Molecular Imager Gel Doc XR+(Bio-Rad) before transferring the protein onto a PVDF membrane. The membrane was blocked in 5% nonfat dry milk in TBS for 1 h, followed by incubation with the primary antibodies: rabbit anti-γ-tubulin (1:1000, A302-631A-M; Bethyl, Montgomery, TX, USA) and mouse anti GCP2 (1:1000, NBP2-21793; Novus Biologicals), in blocking buffer overnight at 4 °C. The membranes were washed three times with TBS-T and then incubated with goat-anti-rabbit (Bio-Rad) or goat-anti-mouse (Cell Signaling, Danvers, MA, USA) (1:5000 dilution) secondary antibodies conjugated to horseradish peroxidase for 1 h. The membranes were washed three times with TBS-T and signals were detected by Clarity Western ECL Substrate (Bio-Rad).

### 2.4. Growth Curve of Patient and Control Lymphoblasts

Each of 2 × 105 patient or control lymphoblasts were seeded into each well of 6-well plates in RPMI 1640 medium supplemented with 10% FBS. The cells were collected from three independent wells and counted by a Corning Cell Counter (CytoSMART Technologies, Eindhoven, The Netherlands) at the indicated time point. The experiment was repeated three times independently.

### 2.5. Isolation of Total RNA, Reverse-Transcription PCR and qPCR

The total RNA was isolated using an RNeasy Plus Mini Kit (Qiagen, Hilden, Germany) and reversed transcribed into cDNA using the iScript Reverse Transcription Supermix (Bio-Rad) according to the respective manufacturer instructions. Real-time PCR was performed using 1 μL of the 1:10 diluted cDNA in a 10 μL of reaction with 500 nM final concentration of gene-specific primers (Supplementary Table S1) along with iQ SYBR Green Supermix (Bio-Rad) in a CFX96 Touch Real-Time PCR Detection System (Bio-Rad). cDNAs were amplified under the following conditions: 40 cycles of 95 °C for 10 s, 55 or 59 °C for 30 s, 72 °C for 30 s and a final melting curve generated in increments of 0.5 °C per plate reading.

### 2.6. Co-Immunoprecipitation

The 293T cells transfected with pcDNA3.1 plus plasmid expressing wild-type, variant TUBG2 or the empty pcDNA3.1 plus plasmid were harvested by trypsinization, rinsed with PBS and frozen at −80 °C. The pellets were resuspended in 50 mM HEPES-KOH pH 7.2, containing 1 mM MgCl_2_, 1 mM EGTA, 100 mM KCl, 0.5 mM GTP and 1% Halt protease inhibitor cocktail (Thermofisher, Waltham, MA, USA). The cells were disrupted by sonication and clarified by centrifugation. Two micrograms of mouse anti DYKDDDK (FLAG) tag antibody (A00187; GenScript, Piscataway, NJ, USA) pre-bound to 50 μL of Dynabeads protein G slurry (Invitrogen), then incubated with 150–260 μg of clarified cell lysate in a total volume of 200 μL with gentle agitation at 4 °C overnight. The bead Ab–Ag complexes were washed by cell pellet suspension buffer supplemented by 0.1% Tween-20 three times, followed by elution one time with NuPAGE LDS sample buffer (Invitrogen) containing 16.7 mM glycine (pH 2.8) and 20 mM DTT at 70 °C for 10 min with vigorous shaking. The immunoprecipitated proteins were separated by 10% TGX Stain-Free acrylamide gel (Bio-Rad) and were Western blotted as described above. The primary antibodies used here were the mouse anti DYKDDDK tag antibody (0.5 μg/mL; GenScript) for FLAG-tagged TUBG2 input and rabbit anti GCP2 (1:1000, PA5-58151; Thermofisher) for both input and immunoprecipitated GCP2. The rabbit anti-γ-tubulin described above was used for the immunoprecipitated recombinant FLAG tagged TUBG2 to avoid the recognition of mouse IgG heavy chain.

## 3. Results

### 3.1. Case Report

**Figure 1:**
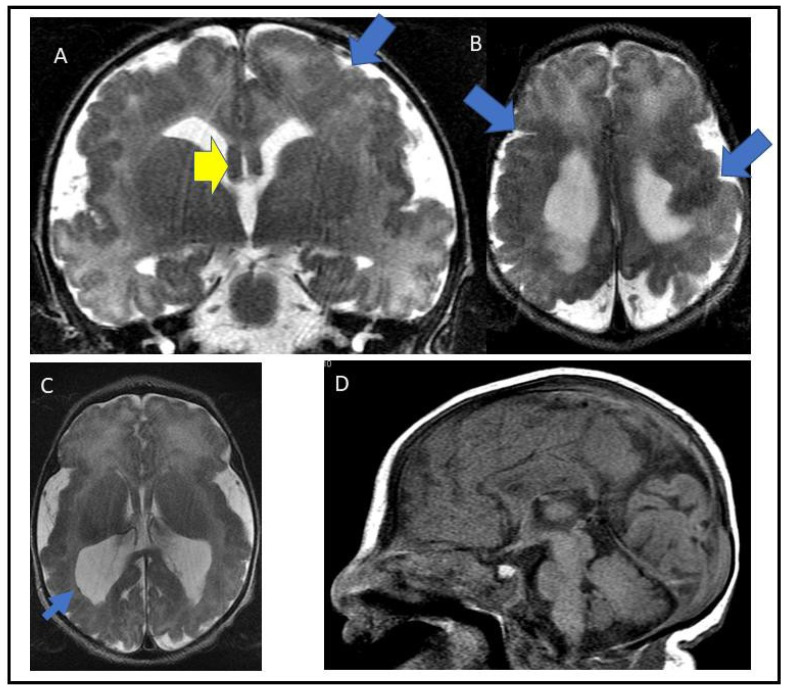
Coronal and axial T2WI show diffuse polymicrogyria with possible close lip schizencephaly in both frontal and left parietal lobes (thick blue arrows; (**A**,**B**)) and a small focus of gray-matter periventricular heterotopia in the left ventricular atrium (thin blue arrow; (**C**)). Colpocephaly with thickening of the bilateral fornixes (thick yellow arrow; (**A**)) were also identified. The hippocampal commissure appears intact. (**D**); Sagittal T1WI shows microcephaly with proportional size of the posterior fossa and supratentorial neural structures. There are deep parietooccipital fissures. The corpus callosum appears to be dysplastic posteriorly with associated colpocephaly.

A newborn male was referred to the Genetics Clinic for an assessment of his complex brain malformation. He was diagnosed prenatally with bilateral perisylvian polymicrogyria with extensive abnormality involving the adjacent parenchyma by fetal MRI at 33 weeks gestation. The pregnancy itself was otherwise uncomplicated. His mother was a healthy 30-year-old G2P0 who had no teratogenic exposures and only took prenatal vita- mins during the pregnancy. He was born at 37 + 4 weeks gestational age via spontaneous vaginal birth. The APGARs were 81 min and 95 min, and no resuscitation was required. His birth weight was 3215 g (75–90th percentile) and the head circumference was 34.1 cm (24th percentile). The neonatal examination was only remarkable for midshaft hypospadias. A neonatal MRI at 2 days of life confirmed diffuse polymicrogyria involving mainly perisylvian regions, parietal, temporal and frontal lobes, as well as colpocephaly with a dysplastic corpus callosum (Figure 1).

At three and half months of age, the patient presented with clinical spasms and developmental regression. He was diagnosed with infantile spasms with an EEG showing hypsarrhythmia. Since that time, his seizures have been refractory to therapy. His current seizure semiologies include epileptic spasms, tonic seizures and focal motor seizures. He has failed vigabatrin, prednisolone, topiramate, valproic acid, lamotrigine, clobazam, rufinamide, ketogenic diet and cannabis. He is not a surgical candidate due to the extensive nature of the polymicrogyria. Vagus nerve stimulation surgery was offered but declined by family. At his last assessment, he was non-dysmorphic in appearance and microcephalic (8 years old; OFC of 49.3 cm; SD—2.2). He was non-verbal, had marked axial hypotonia with increased appendicular tone and a spastic quadriplegia with brisk reflexes. Genetic testing included a microarray (normal) as well as *ADGRG1* sequencing (normal). As no diagnosis for the child’s complex brain malformation was identified, the family was offered participation in research. Free and informed consent was obtained for a research protocol (Care4Rare research; REB#11/04E). Trio-exome sequencing identified a novel variant in *TUBG2* (NM_016437.3 c.747G>A p.(Met249Ile)). The siblings (younger brother and sister) did not carry this variant. The variant has not been observed in the local research database (Care4Rare) and was also not observed in gnomAD [14]. In silico prediction program CADD predicted this to be deleterious (22.8). The gene is not known to be associated with human disease; however, given its de novo status, rarity, and knowledge that the gene belongs to the tubulin family of genes, the variant was considered a candidate in a gene of uncertain significance and thus, functional studies were performed.

### 3.2. Cell Growth

**Figure 2:**
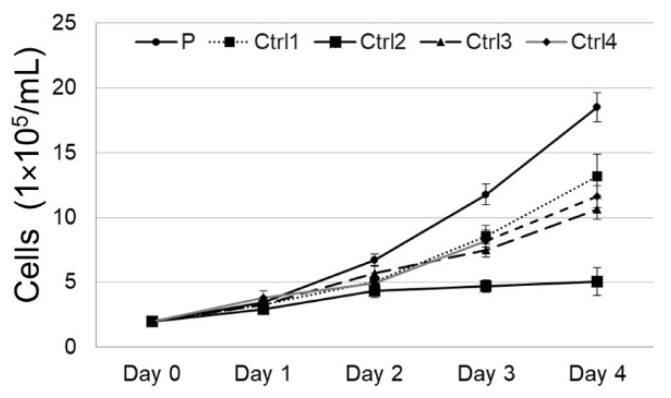
TUBG2 dysregulation leads to increased growth in patient cells. Cell counts for four consecutive days with TUBG2 patient and control cells display that the patient cells reproduce significantly faster than age and sex matched control cells (Day 3 and Day 4; *p* = 0.06 (ns) and *p* = 0.04 respectively).

Cell counts performed showed that the cells cultured from the patient grew significantly (*p* = 0.04; Day 4) faster than controls over a four day period (Figure 2).

### 3.3. TUBG2 Expression and Function: Western Blot and RT-PCR

**Figure 3:**
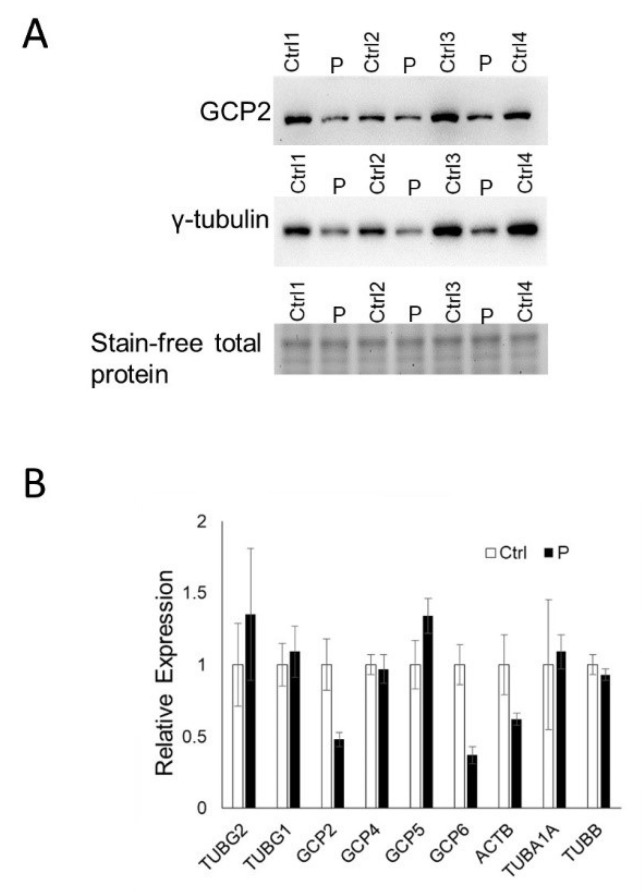
Patient cells display decreased expression of GCP2 and other complex members. (**A**) GCP2 193 and γ-tubulin are decreased in patient cells compared to 4 age-matched, unrelated healthy controls (*p* = 0.06, ns; *p* = 0.05 respectively). Stain-free total protein is included. (**B**) Endogenous mRNA expression of TUBG2 is not statistically different in patient versus matched controls, and there is variable expression of other complex members with some being unaltered (TUBG1, GCP4, GCP5, TUBA1A, TUBB, ACTB1) and others are decreased (GCP2 (*p* = 0.06, ns), GCP6 (*p* = 0.01). Statistical comparisons are made with a two-tailed Student *t*-test.

**Figure 4:**
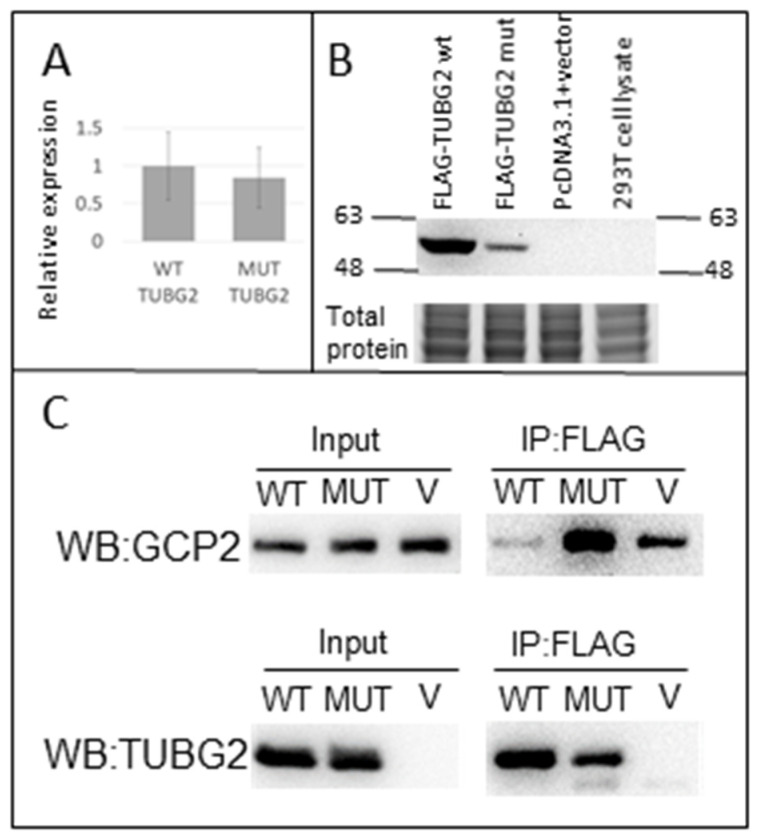
(**A**) shows the mRNA expression of exogenous wild-type (WT) and exogenous mutant (MUT). They are not significantly different (*p* = n.s.). (**B**) This shows the decreased protein expression of the recombinant mutant protein compared to recombinant WT protein. (**C**) GCP2 and TUBG2 interaction is increased due to the p.(Met249Ile) variant. Co-IP in cells transfected with either WT, MUT or empty vector demonstrates that the TUBG2-GCP2 interaction is increased due to the MUT vector versus WT vector.

A Western blot analysis for endogenous γ-tubulin showed that the total γ-tubulin level was lower in the patient lymphoblasts compared with controls (*p* = 0.05; Figure 3A). Humans possess two genes encoding γ-tubulin, TUBG1 and TUBG2, which have more than >94% sequence similarity; there are no commercially available antibodies that can distinguish them. To investigate the effect of the TUBG2 substitution at the protein level, we expressed the recombinant wild type and mutant TUBG2 with FLAG tags in 293T cells. The 293T cells expressed lower levels of TUBG2 mutant compared with the TUBG2 wild-type by Western blot (Figure 4B). The TUBG2 mutant wild-type exhibited similar mRNA levels for both recombinant and endogenous TUBG2 in RT-PCR (Figure 3B and Figure 4A). The observations indicate the p.(Met249Ile) substitution may affect TUBG2 protein stability.

### 3.4. RT-PCR of TUBG1

We also analyzed the mRNA level of another γ-tubulin gene, *TUBG1*. There was no difference in the *TUBG1* mRNA level between the patient and control lymphoblasts (Figure 3B) consistent with the lack of a compensative response from this gene.

### 3.5. RT-PCR of γ-Tubulin Complex Proteins

In humans, γ-tubulin assembles γ-tubulin complex proteins (GCPs) into two main γ-tubulin complexes (γ-TuCs). We quantitated the mRNA level of GCP2, GCP4, GCP5 and GCP6 in the patient and control lymphoblasts. There was no difference in the mRNA level for these genes, except for GCP6 (*p* = 0.01) and possibly GCP2 that was trending towards significance (*p* = 0.06) (Figure 3B). Our RT-PCR analysis also showed no differences in mRNA level for some cytoskeleton genes, ACTB and TUBA1A (Figure 3B).

### 3.6. Co-Immunoprecipitation

An interaction between recombinant TUBG2 and GCP2 was detected and was increased with a mutant protein compared to WT (Figure 4C).

## 4. Discussion

The evidence required to declare a disease–gene association is rigorous and typically requires the reporting of several individuals presenting with similar phenotype and deleterious variant(s) in a shared gene, functional studies, model systems and, importantly, independent replication over time [15]. Here, we report the first evidence in favour of *TUBG2* as a potential disease gene associated with polymicrogyria and its sequelae of microcephaly, dysplastic corpus callosum, intellectual disability and seizures hat are refractory to treatment. The genetic evidence in favour of this hypothesis is a missense variant that has not been seen before in available control databases, it is de novo and in silico programs predict it to be deleterious.

*TUBG2* is a compelling disease gene candidate based on several lines of evidence. It is highly homologous to *TUBG1*, a gene known to be associated with complex brain mal-formations [16,17,18]. TUBG1 shares over 97% of its amino acids with TUBG2 and the two genes share similar exon/intron boundaries and arose from a duplication event [19]. While different than the ubiquitously expressed *TUBG1*, *TUBG2* expression is restricted to the brain and consistent with the gene responsible for a complex brain malformation. Both TUBG1 and TUBG2 are concentrated at centrosomes during interphase and mitosis [19]. *TUBG1*-associated brain malformations appear to result from a gain-of-function mechanism as only missense variants have been reported to date (*n* = 10) associated with brain malformations [16,18,20]. Mice that are deficient n Tubg2 have a normal phenotype, which suggests that a loss of function is not responsible or an obvious phenotype associated with this mechanism [19]. This does differ from Tubg1, where mice embryos deficient in Tubg1 do not progress past morula/blastocyst stage [19].

Evidence from our own functional work is also consistent with a dysregulation of growth and differences in the ability of TUBG2 to interact with its binding partners (GCP2; Figure 4).

Tubulopathies are associated with a wide range of brain malformations that include lissencephaly, polymicrogyria and dysgenesis of the corpus callosum. The genes include *TUBA1A*, *TUBB2A*, *TUBA8*, *TUBB2B*, *TUBB3*, *TUBB5* and *TUBG1* [9]. The phenotypes associated with these reported individuals share similar features to our patient and they have ID, epilepsy and microcephaly. *TUBG1* is associated with pachygyria of the occiput and parietal lobes [16,18]. It is also noted to have normal cerebellum, basal ganglia and brainstem, again similar to our patient.

In closing, we present initial evidence for *TUBG2* as a novel disease gene. While compelling, additional reports of individuals with a de novo variation and similar phenotype are required to confirm the disease association of *TUBG2*.

## Data Availability

Not applicable.

## Supplementary Materials

The following supporting information can be downloaded at: https://www.mdpi.com/article/10.3390/genes14010108/s1, Table S1: TUBG2-specific primers.
