# Supplementary material for: A De Novo Missense Variant in TUBG2 in a Child with Global Developmental Delay, Microcephaly, Refractory Epilepsy and Perisylvian Polymicrogyria
Source: Genes (Basel). 2022 Dec 29;14(1):108. doi: 10.3390/genes14010108 (PMC9859306; doi:10.3390/genes14010108)
Supplement: Supplementary file 1 [file genes-14-00108-s001.zip › genes-1989470-supplementary.pdf]

Supplementary Table S1. Quantitative real-time PCR primers

Gene	Sequence	Annealing temperature (℃)
TUBG2	Forward:5'-TCAAGAGGCGAAGAGAGCG-3'	59
	Reverse:5'-TCCAGAACTCGAACCCAATCTG-3'	
TUBG1	Forward:5'-CAACGCCGGTGCCTGA-3'	59
	Reverse:5'-TGCTCATCGTCTGCCTGGTA-3'	
ACTB	Forward:5'-ACAGAGCCTCGCCTTTGCC-3'	55
	Reverse:5'-GATATCATCATCCATGGTGAGCTGG-3'	
TUBB	Forward:5'-TGTGGTACGGAAGGAGGTCGAT-3'	55
	Reverse:5'-CGGCTGTCTTGACATTGTTGG-3'	
TUBA1A	Forward:5'-GTCGCGCTGTAAGAAGCAAC-3'	55
	Reverse:5'-GCACTCACGGAAAGAAAAGAGC-3'	
GCP2	Forward:5'-TGATGTTCGTGCCTCAGAGC-3'	55
	Reverse:5'-TAGACCTCAGCCCCATCTCC-3'	
GCP4	Forward:5'-CCATGTGCAACAGCAGGATCA-3'	55
	Reverse:5'-AGTGCTTGGCGATAAGGCTG-3'	
GCP5	Forward:5'-GGGCACTTGGCGAATGGA-3'	55
	Reverse:5'-CCAGCCATGAGTGACAACGC-3'	
GCP6	Forward:5'-GGGCTGGCTATTAAGGTCCC-3'	55

	Reverse:5'-GGAGTCAAGTTGGACGCTGA-3'	
GAPDH	Forward:5'-CACTAGGCGCTCACTGTTCT-3'	55
	Reverse:5'-GACCAAATCCGTTGACTCCG-3'	
TUBG2-FLAG	Forward:5'-GGGAGGTTGTTCAGGAGCTC-3'	55
	Reverse:5'-CTTATCGTCGTCATCCTTGTAATC-3'	
